# Barcoding of Arrow Worms (Phylum Chaetognatha) from Three Oceans: Genetic Diversity and Evolution within an Enigmatic Phylum

**DOI:** 10.1371/journal.pone.0009949

**Published:** 2010-04-01

**Authors:** Robert M. Jennings, Ann Bucklin, Annelies Pierrot-Bults

**Affiliations:** 1 Biology Department, University of Massachusetts at Boston, Boston, Massachusetts, United States of America; 2 Marine Sciences Department, University of Connecticut at Avery Point, Groton, Connecticut, United States of America; 3 Zoölogisch Museum Amsterdam, Amsterdam, The Netherlands; Northeastern University, United States of America

## Abstract

Arrow worms (Phylum Chaetognatha) are abundant planktonic organisms and important predators in many food webs; yet, the classification and evolutionary relationships among chaetognath species remain poorly understood. A seemingly simple body plan is underlain by subtle variation in morphological details, obscuring the affinities of species within the phylum. Many species achieve near global distributions, spanning the same latitudinal bands in all ocean basins, while others present disjunct ranges, in some cases with the same species apparently found at both poles. To better understand how these complex evolutionary and geographic variables are reflected in the species makeup of chaetognaths, we analyze DNA barcodes of the mitochondrial cytochrome oxidase *c* subunit I (COI) gene, from 52 specimens of 14 species of chaetognaths collected mainly from the Atlantic Ocean. Barcoding analysis was highly successful at discriminating described species of chaetognaths across the phylum, and revealed little geographical structure. This barcode analysis reveals hitherto unseen genetic variation among species of arrow worms, and provides insight into some species relationships of this enigmatic group.

## Introduction

Arrow worms (Phylum Chaetognatha) comprise over 120 species, all of which inhabit marine environments and exhibit hermaphroditic reproduction. Although there are fewer species in this phylum than in many others, chaetognaths can be numerically abundant in many pelagic environments [1], and their grasping hooks, rows of strong teeth, and transparent bodies make them excellent predators in many marine food webs.

Despite knowledge of chaetognaths extending back to at least the eighteenth century (the first description was by Slabber in 1778), taxonomic affinities of the phylum remain enigmatic. Although fossils are known as far back as the early Cambrian [2], the generally poor preservation of chaetognaths has frustrated attempts to reconstruct their evolutionary history. Chaetognaths appear to have a relatively simple, conserved body plan, with few complex internal structures. However, variation in morphological characters—e.g. position of lateral fins, morphology of tail fins, organization of teeth and grasping hooks—is often a matter of degree rather than of sharp contrast, making classification difficult [3]. Indeed, the seemingly simple morphology of arrow worms belies an underlying mix of features synapomorphic to chaetognaths and features shared with other phyla, complicating placement at even the most basic levels of metazoan organization. Reflective of this complexity, taxonomists have variably placed chaetognaths as basal members of protostomes or deuterostomes [4]–[6] or even outside the coelomate metazoans [7]. Although molecular phylogenetic analyses tend to support placement within the protostomes [6], [8]–[10], alternative arrangements are still advanced.

Although fewer studies have focused on the relationships among the species and proposed families within the Chaetognatha, they too reflect a history of revision. After Tokioka's reorganization of the early chaetognath classification [11], morphological taxonomy has advanced a succession of alternative schemes [12]. For instance, the genus *Sagitta*, which contains some 60 species, has also been considered a family [13]; while this relative placement reflects the Linnaean classification system and is therefore somewhat arbitrary, it does highlight the current uncertainly in timing and driving forces of speciation in Chaetognatha. Morphological identification of arrow worms requires significant training and expertise, and delineating species (that is, identifying monophyletic taxa) has often been difficult, even for experienced taxonomists.

Biogeographical data further complicate our understanding of species structure in chaetognaths. Although many species exhibit large ranges, encompassing similar latitudinal bands in all major oceans [14], chaetognaths can also be accurate indicators of regional water masses and depth layers [15]. Species such as *Sagitta setosa* often exhibit disjunct distributions [12], [16], which raise questions as to whether the morphological variation seen between populations has a genetic basis. Finally, species and/or groups of related species exhibit patterns of distribution that may reflect their history of evolution and speciation. For instance, the cold-water species *Sagitta maxima* exhibits submergence (i.e. a shift into deeper waters) in subtropical and tropical zones. More intriguing are the similar distributions of two groups of chaetognaths, each containing three species (*S. marri, S. zetesios*, *S. planctonis, and S. gazellae, S. maxima, S. lyra*). The first species in each triplet is found in Antarctic waters, the second shows a bipolar distribution with submergence towards the equator, and the third is subtropical [15], [17]. It is not known whether this latitudinal series of distributions reflects the speciation history of the triplets either northwards or southwards, or whether it is an ecological grouping only.

The complex morphological and geographical associations of chaetognaths present a situation in which DNA barcoding [18] can offer significant insight. Analysis of the patterns of DNA sequence diversity at the mitochondrial cytochrome oxidase *c* subunit I (COI) gene, when combined with known morphological associations of established species, results in a fuller understanding not only of the cohesion of well-known taxa, but also the range of variation contained within them. Barcoding using COI has been effective in revealing previously unknown patterns of genetic diversity in terrestrial systems (e.g. [19]–[21]) and marine systems (e.g. fish [22], chitons [23], and crustaceans [24]). While nuclear rRNA genes are frequently used in similar investigations, their resolution is typically taxonomically deeper than the species level crucial to species discrimination with COI. Further, the ribosomal genes copies in chaetognaths appear to be split into two highly divergent “classes” [3]; [25] whose paralog vs. pseudogene status remains unclear. This possibly non-homogeneous duplication of nuclear ribosomal genes complicates their use in genetic analyses. This study presents DNA barcodes for 52 specimens of 14 species of chaetognaths collected from the Atlantic and Southern Oceans. These collections are part of an ongoing barcoding effort of the Census of Marine Zooplankton (CMarZ) and the Mid-Atlantic Ridge Ecology group (MAR-ECO), two field projects of the Census of Marine Life (CoML).

## Materials and Methods

**Figure 1:**
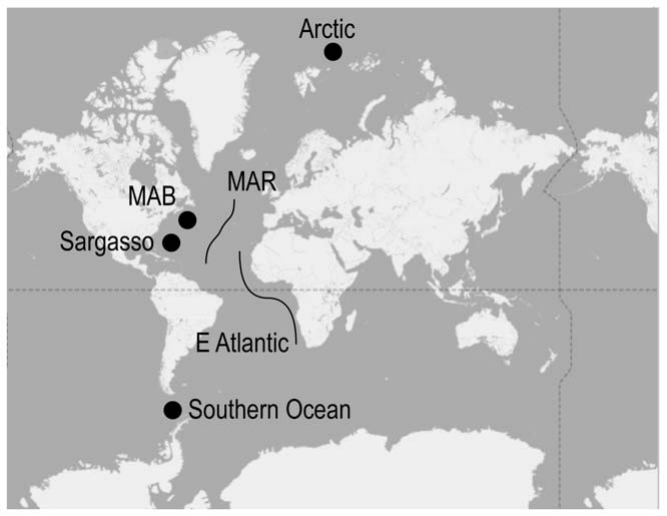
Map showing locations of cruises and material collected in this study.

Chaetognaths sequenced in this project were collected on six cruises (Figure 1). A cruise collected zooplankton from the waters west of the Antarctic Peninsula on board the *R/V N.B. Palmer* in 2002 (NBP0202). Four cruises sampled waters in the Atlantic: the R/V *G.O. Sars* to the northern Mid-Atlantic Ridge (MAR) in summer 2004 (SARS_2004110), the R/*V R.H. Brown* in April 2006 (RHB0603) to the Sargasso Sea (Northwest Atlantic), the *R/V Delaware II* in November 2006 (DL0616) to the Mid-Atlantic Bight (MAB), and the FS *Polarstern* along the eastern boundary of the Atlantic (Canary Islands to South Africa) in November 2007 (PS-ANT-XXIV/1). Finally, the FS *Polarstern* collected zooplankton from the Arctic Ocean north of Europe in summer 2007 (PS-ARK-XXII/2).

For some specimens, DNA extraction, PCR amplification, and sequencing took place during the cruise; other specimens were analyzed at the University of Connecticut. Procedures and equipment were the same for all specimens. Vouchered material was preserved in acetone (MAR and Southern Ocean cruises) or 95% ethanol (Northwest Atlantic, MAB, Eastern Atlantic, and Arctic cruises). The voucher consisted of at least one additional individual taken from the same net tow, or as necessary, a minimal amount of excised tissue of an individual specimen was removed for DNA extraction and the remainder retained as the voucher. All vouchers are therefore paragenophores (*sensu* [26]). Photographs were taken of specimens before dissection when possible. Vouchers and images are maintained by CMarZ at the University of Connecticut, USA. Collection information and species identifications are summarized in Table 1.

**Table 1:** Species identity and collection information for barcoded chaetognaths.

Species	Voucher no.	Geographic location	Cruise	Collection date	Station	Latitude Longitude	GenBank Acc. No.
Eukrohnia bathyantarctica	Ch03.1.1	Northern MAR	Sars_2004110	13-Jun-2004	6	57.15 N 31.10 W	GQ368374
E. bathyantarctica	Ch03.1.2	Northern MAR	Sars_2004110	13-Jun-2004	6	57.15 N 31.10 W	GQ368375
E. bathyantarctica	Ch03.1.6	Northern MAR	Sars_2004110	13-Jun-2004	6	57.15 N 31.10 W	GQ368376
E. bathyantarctica	Ch03.1.7	Northern MAR	Sars_2004110	13-Jun-2004	6	57.15 N 31.10 W	GQ368377
E. bathyantarctica	Ch03.1.8	Northern MAR	Sars_2004110	13-Jun-2004	6	57.15 N 31.10 W	GQ368378
E. bathyantarctica	Ch03.1.9	Northern MAR	Sars_2004110	13-Jun-2004	6	57.15 N 31.10 W	GQ368379
E. bathyantarctica	Ch03.1.10	Northern MAR	Sars_2004110	13-Jun-2004	6	57.15 N 31.10 W	GQ368380
E. bathyantarctica	Ch12.1.1	Northern MAR	Sars_2004110	13-Jun-2004	6	57.15 N 31.10 W	GQ368381
E. bathyantarctica	Ch12.1.2	Northern MAR	Sars_2004110	13-Jun-2004	6	57.15 N 31.10 W	GQ368382
E. bathyantarctica	Ch12.1.3	Northern MAR	Sars_2004110	13-Jun-2004	6	57.15 N 31.10 W	GQ368383
E. fowleri	Ch02.1.1	Northern MAR	Sars_2004110	13-Jun-2004	6	57.15 N 31.10 W	GQ368384
E. fowleri	Ch02.1.2	Northern MAR	Sars_2004110	13-Jun-2004	6	57.15 N 31.10 W	GQ368385
E. fowleri	Ch02.1.3	Northern MAR	Sars_2004110	13-Jun-2004	6	57.15 N 31.10 W	GQ368386
E. fowleri	Ch02.3.1	NE Atlantic	PS-ANT-XXIV/1	8-Nov-2007	2	11.68 N 20.42 W	GQ368387
E. hamata	Ch19.7.1	SE Atlantic	PS-ANT-XXIV/1	20-Nov-2007	7	23.24 S 8.24 E	GQ368388
E. hamata	Ch19.8.1	SE Atlantic	PS-ANT-XXIV/1	21-Nov-2007	8	25.60 S 9.74 E	GQ368389
E. hamata	Ch19.9.3	SE Atlantic	PS-ANT-XXIV/1	17-Nov-2007	6	13.42 S 0.65 E	GQ368390
E. macroneura	Ch19.6.1	NE Atlantic	PS-ANT-XXIV/1	11-Nov-2007	3	3.51 N 14.00 W	GQ368391
E. macroneura	Ch19.6.2	NE Atlantic	PS-ANT-XXIV/1	11-Nov-2007	3	3.51 N 14.00 W	GQ368392
E. macroneura	Ch19.6.3	NE Atlantic	PS-ANT-XXIV/1	11-Nov-2007	3	3.51 N 14.00 W	GQ368393
Heterokrohnia mirabilis	Ch30.1.2	SE Atlantic	PS-ANT-XXIV/1	17-Nov-2007	6	13.16 S 0.32 W	GQ368394
Heterokrohnia sp.	Ch26.1.1	Arctic	PS-ARK-XXII/2	8-Aug-2007	260	84.49 N 36.14 E	FJ602474
Sagitta bipunctata	Ch22.1.1	NW Atlantic	RHB0603	25-Apr-2006	5	14.00 N 55.00 W	GQ368396
S. bipunctata	Ch22.1.2	NW Atlantic	RHB0603	25-Apr-2006	5	14.00 N 55.00 W	GQ368397
S. bipunctata	Ch22.2.1	NW Atlantic	RHB0603	25-Apr-2006	5	14.00 N 55.00 W	GQ368398
S. enflata	Ch15.1.1	NW Atlantic	RHB0603	25-Apr-2006	5	14.00 N 55.00 W	GQ368399
S. enflata	Ch15.1.2	NW Atlantic	RHB0603	25-Apr-2006	5	14.00 N 55.00 W	GQ368400
S. enflata	Ch15.2.1	MAB	DL0616	6-Nov-2006	2	39.14 N 72.97 W	GQ368401
S. helenae	Ch16.1.1	NW Atlantic	RHB0603	25-Apr-2006	5	14.00 N 55.00 W	GQ368402
S. helenae	Ch16.2.1	NW Atlantic	RHB0603	25-Apr-2006	5	14.04 N 54.89 W	GQ368403
S. helenae	Ch16.3.1	NW Atlantic	RHB0603	25-Apr-2006	5	14.04 N 54.89 W	GQ368404
S. lyra	Ch07.1.1	Northern MAR	Sars_2004110	1-Jul-2004	36	41.48 N 28.42 W	GQ368405
S. lyra	Ch07.1.2	Northern MAR	Sars_2004110	1-Jul-2004	36	41.48 N 28.42 W	GQ368406
S. lyra	Ch07.1.5	Northern MAR	Sars_2004110	1-Jul-2004	36	41.48 N 28.42 W	GQ368407
S. lyra	Ch07.1.6	Northern MAR	Sars_2004110	1-Jul-2004	36	41.48 N 28.42 W	GQ368408
S. lyra	Ch07.5.3	NE Atlantic	PS-ANT-XXIV/1	5-Nov-2007	1	24.68 N 20.75 W	GQ368409
S. lyra	Ch07.5.2	NE Atlantic	PS-ANT-XXIV/1	5-Nov-2007	1	24.68 N 20.75 W	GQ368410
S. lyra	Ch07.5.1	NE Atlantic	PS-ANT-XXIV/1	5-Nov-2007	1	24.68 N 20.75 W	GQ368411
S. marri	Ch18.1.1	Southern Ocean	NBP0202	9-May-2002	89	68.81 S 76.98 W	GQ368412
S. marri	Ch18.1.2	Southern Ocean	NBP0202	9-May-2002	89	68.81 S 76.98 W	GQ368413
S. marri	Ch18.1.3	Southern Ocean	NBP0202	9-May-2002	89	68.81 S 76.98 W	GQ368414
S. planctonis	Ch10.1.1	Northern MAR	Sars_2004110	28-Jun-2004	30	42.95 N 29.30 W	GQ368415
S. planctonis	Ch10.1.2	Northern MAR	Sars_2004110	28-Jun-2004	30	42.95 N 29.30 W	GQ368416
S. planctonis	Ch10.1.3	Northern MAR	Sars_2004110	28-Jun-2004	30	42.95 N 29.30 W	GQ368417
S. sibogae	Ch21.1.1	NW Atlantic	RHB0603	25-Apr-2006	5	14.04 N 54.89 W	GQ368418
S. sibogae	Ch21.1.2	NW Atlantic	RHB0603	25-Apr-2006	5	14.04 N 54.89 W	GQ368419
S. sibogae	Ch21.1.3	NW Atlantic	RHB0603	25-Apr-2006	5	14.04 N 54.89 W	GQ368420
S. sibogae	Ch21.2.1	NW Atlantic	RHB0603	25-Apr-2006	5	14.00 N 55.00 W	GQ368421
S. zetesios	Ch11.1.1	Northern MAR	Sars_2004110	13-Jun-2004	6	57.15 N 31.10 W	GQ368422
S. zetesios	Ch11.1.2	Northern MAR	Sars_2004110	13-Jun-2004	6	57.15 N 31.10 W	GQ368423
S. zetesios	Ch11.1.3	Northern MAR	Sars_2004110	13-Jun-2004	6	57.15 N 31.10 W	GQ368424
S. zetesios	Ch11.2.1	Northern MAR	Sars_2004110	13-Jun-2004	6	57.15 N 31.10 W	GQ368425

For all preserved, identified specimens, DNA analysis proceeded as follows. Up to 25 mm^3^ of tissue from single arrow worms was dissected using sterile techniques and DNA was extracted with the DNEasy DNA Extraction Kit (Qiagen). PCR amplification of the COI barcode region employed primers LCO-1490 (5′-GGTCAACAAATCATAAAGATATTGG-3′) and HCO-2198 (5′-TAAACTTCAGGGTGACCAAAAAATCA-3′) from [27], in 50 µL PCRs consisting of 1x GoTaq Flexi buffer (Promega, Madison, WI USA), 2.5 mM MgCl_2_, 2 pmol dNTPs, 1.2 pmol of each primer, approximately 50 ng extracted DNA template, and 1U of Taq polymerase (Promega). The PCR protocol was as follows: initial denaturation, 95°C for 5 min.; 35 cycles of (95°C for 30 sec., 50°C for 45 sec., 72°C for 1 min); final extension, 72°C for 5 min. Products were purified using the QIAquick PCR Purification Kit (Qiagen, Valencia, CA USA). Sequencing reactions were performed using BigDye Terminators v3.1, purified via ethanol precipitation, and run on an ABI 3130 Automated Sequencer.

Forward and reverse sequences for each individual were assembled in Sequencher (GeneCodes, Inc., Ann Arbor, MI USA) and manually edited. All sequences were compared to the GenBank database using BLAST [28], and to a database of all zooplankton barcodes obtained in the laboratory (Bucklin et al. unpublished). Edited DNA sequences were exported into BioEdit and translated to inferred amino acid sequences to verify that they translated correctly. Once verified, the COI sequences were aligned as amino acids using the CLUSTAL algorithm [29] in BioEdit, and returned to DNA format. This alignment was manually edited for consistency and to remove primer sequences. The final dataset contained sequences for 52 specimens of 14 species of chaetognaths. For reference, three COI sequences of *Sagitta bedoti* from [16] were added from GenBank. Sequences produced in this project were deposited in the BARCODE section of GenBank along with georeferenced metadata (Accession Numbers GQ368374-GQ368425).

To investigate the levels of genetic variation within and between chaetognath species, pairwise Kimura 2-parameter distances (K2P; [30]) were computed in MEGA 4 [31], with gap positions ignored on a pairwise basis. These distances were hierarchically tabulated within each species, and between species within each genus. Because the sequence dataset contained only two genera from the same family (*Eukrohnia* and *Heterokrohnia*), comparisons between genera within the family were not tabulated.

To investigate the evolutionary history of COI sequences in chaetognaths, a model of DNA sequence evolution was chosen using MrModeltest v2 [32] under Akaike's Information Criterion (AIC). The general time-reversible model (GST) was selected, with an estimated proportion of DNA sites invariant (I), and mutation rates among sites following a gamma distribution (G). This GTR+I+G model was then used to generate a Bayesian and a maximum likelihood (ML) gene tree. The Bayesian tree was obtained with MrBayes 3.1.2 [33], with the search conducted 100,000 iterations at a time, continuing until the average standard deviation of split frequencies approached its asymptote (roughly 0.01 after 400,000 generations). The collection of trees produced at this point was pruned heuristically by viewing the output of likelihood scores in MrBayes, and only trees near the optimum likelihood score were retained using the appropriate burn-in criterion. The final sample contained 2000 trees, on which posterior probabilities (PP) were calculated. To construct the ML tree, the hill-climbing algorithm of [34] was performed online via the PHYML web server [35], using the default options, the chosen GTR+I+G model, and a starting tree made by neighbor joining. For consistency with MrBayes, in which the form of the molecular model is specified but parameters are estimated, only the model form was specified in PHYML. Support for nodes in the tree was assessed using the approximate likelihood ratio test (aLRT, [36]) as implemented in PHYML.

## Results

**Figure 2:**
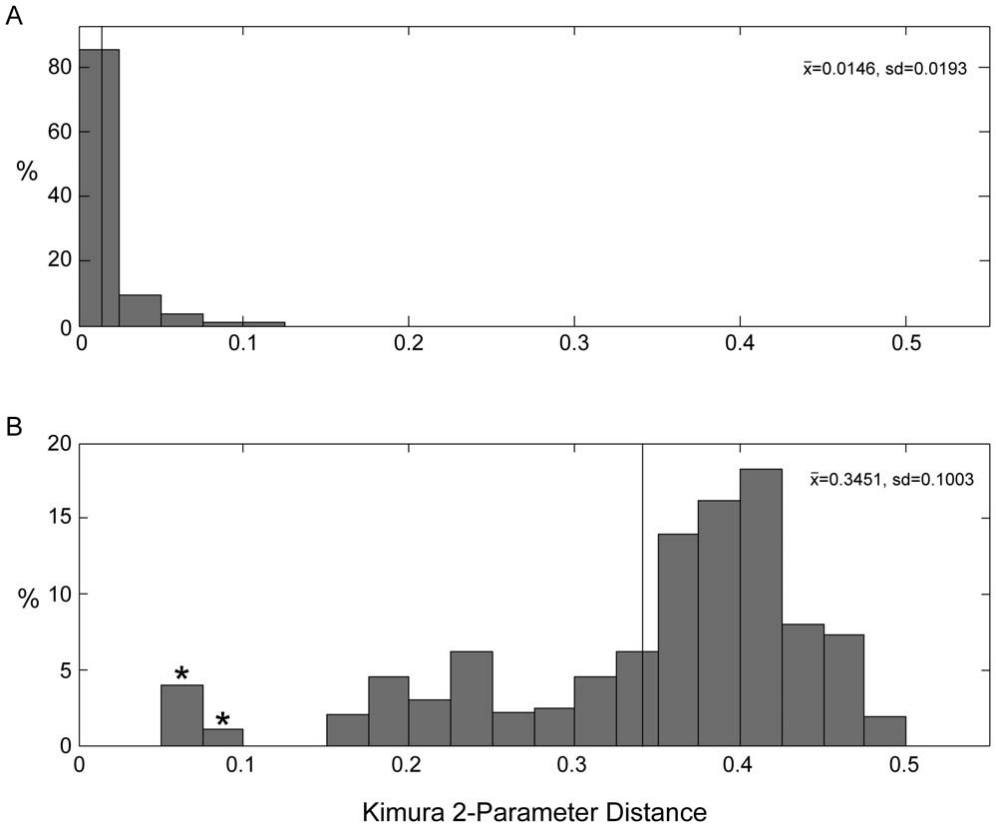
Hierarchical histograms of pairwise Kimura 2-Parameter (K2P) distances between specimens. Vertical lines show mean pairwise distance at each level. Asterisks mark outlier values discussed in the Results. A, K2P distances within species. B, K2P distances between species within each genus.

Hierarchical comparison of K2P distances at different taxonomic levels revealed disjunct distributions in sequence similarity within vs. between species (Figure 2A,B). The average proportion of difference in sequences within species was 0.0146±0.0193 (mean±SD), whereas mean distance between species within a genus was over an order of magnitude larger, 0.345±0.100. The only overlap between these distributions results from comparisons of *Eukrohnia hamata* and *E. bathyantarctica* (K2P distances of 0.06–0.08).

**Figure 3:**
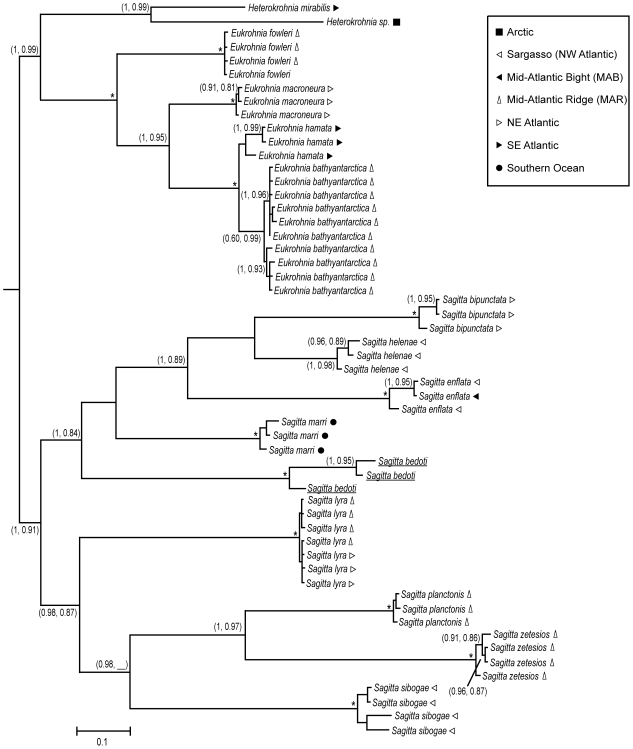
Gene tree for COI, showing topology and branch lengths from Bayesian analysis. Pairs of numbers in parentheses are support values, given as (Bayesian posterior probabilities, approximate Likelihood Ratio Test support), with asterisks indicating maximum support of (1.00, 1.00), and blanks indicating topologies not recovered in that analysis. Scale bar denotes distance along branches. Underlined sequences were obtained from GenBank. Symbols following species names depict sampling location.

The optimal gene trees produced by Bayesian and ML searches showed nearly identical topology, in which the tip branches within species were short, and species were separated by much longer branches (Figure 3). Sequences clustered strongly by species in all cases. Although the nodes separating *Sagitta* spp. from all others *(Heterokrohnia* and *Eukrohnia* spp.) were well supported in the Bayesian analysis (both PP = 1.00), they were not well supported by ML (71% and <50%). Most other internal nodes were moderately supported by both analyses, or strongly supported by only the Bayesian analysis.

## Discussion

Barcode analysis of chaetognaths was extremely successful in diagnosing established species based on COI gene sequence, in that sequences clustered by species in all cases. Given the difficulty in diagnosing species from morphological features, especially in ethanol-preserved material, the high accuracy of barcode analysis presents a very useful tool to aid identification of known species. The comparatively short branch (and small K2P distances) between *E. hamata* and *E. bathyantarctica* may mean these are a young species pair, or that regional variants of a single species have been mistaken for separate species.

The average K2P distance within species for chaetognaths, 0.0145, was on the high end of values computed for other taxa: recent barcoding work has reported intraspecific mean K2P distances of 0.00460 (decapods, [24]), 0.00740 (gammarid amphipods, [24]), 0.0100–0.0200 (13,000 species pairs, [37]), and 0.00390 (fish, [22]). The average distance between the species within each genus for the present dataset, 0.345, was considerably larger than for these same taxa (0.170, 0.0.250, 0.110, and 0.099 respectively), and reflects the high diversity of *Sagitta*. Although not directly comparable to the K2P distances reported here, uncorrected p-distances of 6.30±2.74% (mean±SD) within *Sagitta setosa*, 2.08±0.95% within *S. bedoti*, and maximum-likelihood corrected distances of 77.7±3.45% between the two species have been reported [38]. These comparisons all indicate that most chaetognath species seem to have diverged long ago, and have undergone comparatively less divergence since. The disjunct distributions of K2P distances imply that barcode analysis can also alert taxonomists to genetically distinct lineages that warrant further morphological examination.

Although all barcodes for a given species in this dataset tended to be from the same locality, the genetic variation seen within species showed little association with geography. Most species (e.g. *S. bipunctata, S. helenae, E. hamata*) exhibited at least one barcode separated from the others by a longer branch, even though all were from the same location. In *S. lyra*, there was a weak clustering of two clades, but there was no separation between the central Atlantic (i.e. MAR) and the Northeast Atlantic. The presence of significant genetic diversity without geographic structure could imply reproductive mixing across the portion of the range represented in these specimens, or insufficient time for lineage sorting in isolated populations. More thorough barcoding of species throughout their ranges will be required to address the issue of phylogeography.

Although the COI barcodes did not resolve the branching order of the “paired triplet” species, preliminary analysis suggests that nearly complete sequences of the nuclear large ribosomal subunit (28S) will have the power to address this question (Jennings *et al.* unpublished data). Existing partial Class I sequences [39] contain insufficient variation to obtain robust branching order; however, if the preliminary patterns from full Class I 28S can be confirmed by more complete sequencing, they should shed light on this interesting evolutionary history.

On the whole, the chaetognath barcodes indicate a complex history of speciation and evolution. The lack of correlation between location and genetic similarity underscores this complexity, and the potential for genetic mixing over large distances in chaetognaths. At least for the species in the present analysis, COI barcode analysis was a highly successful and accurate tool for species confirmation, in that all species barcoded to date displayed readily distinguishable COI sequences, with lower divergence within species. Given the difficulty in identifying chaetognaths, particularly from suboptimally preserved material, barcoding of uncertain specimens and comparison to known specimens should greatly assist taxonomists in morphological identifications. More complete barcoding of species across their ranges promises to further elucidate the patterns of genetic diversity of this enigmatic group.

## References

[1] Bone Q, Kapp H, Pierrot-Bults AC (1991). The biology of chaetognaths..

[2] Vannier J, Steiner M, Renvoise E, Hu S-X, Casanova J-P (2007). Early Cambrian origin of modern food webs: evidence from predator arrow worms.. Proceedings of the Royal Society of London B.

[3] Telford MJ, Holland PWH (1997). Evolution of 28S ribosomal DNA in chaetognaths: duplicate genes and molecular phylogeny.. Journal of Molecular Evolution.

[4] Ghirardelli E (1968). Some aspects of the biology of the chaetognaths.. Advances in Marine Biology.

[5] Marlétaz F, Martin E, Perez Y, Papillon D, Caubit X (2006). Chaetognath phylogenomics: a protostome with deuterostome-like development.. Current Biology.

[6] Halanych KM (1996). Testing hypotheses of chaetognath origins: long branches revealed by 18S ribosomal DNA.. Systematic Biology.

[7] Telford MJ, Holland PWH (1993). The phylogenetic affinities of the chaetognaths: a molecular analysis.. Molecular Biology and Evolution.

[8] Telford MJ (2004). Affinity for arrow worms.. Nature.

[9] Papillon D, Perez Y, Caubit X, Le Parco Y (2004). Identification of chaetognaths as protostomes is supported by the analysis of their mitochondrial genome.. Molecular Biology and Evolution.

[10] Matus DQ, Copley RR, Dunn CW, Hejnol A, Eccleston H (2006). Broad taxon sampling and gene sampling indicate that chaetognaths are sister to lophotrochozoans.. Current Biology.

[11] Tokioka T (1965). The taxonomical outline of Chaetognatha.. Publications of the Seto Marine Biological Laboratory.

[12] Peijnenburg KTCA, Fauvelot C, Breeuwer JAJ, Menken SBJ (2006). Spatial and temporal genetic structure of the planktonic *Sagitta setosa* (Chaetognatha) in European seas as revealed by mitochondrial and nuclear DNA markers.. Molecular Ecology.

[13] Bieri R, Bone Q, Kapp H, Pierrot-Bults AC (1991). Systematics of the Chaetognatha.. The biology of chaetognaths.

[14] van der Spoel S, Pierrot-Bults AC (1973). Zoogeography and Diversity of Plankton..

[15] Pierrot-Bults, AC (2008). A short note on the biogeographic patterns of the Chaetognatha fauna in the North Atlantic.. Deep-Sea Research Part II.

[16] Peijnenburg KTCA, Breeuwer JAJ, Pierrot-Bults AC, Menken SBJ (2004). Phylogeography of the planktonic chaetognath *Sagitta setosa* reveals isolation in European seas.. Evolution.

[17] Pierrot-Bults, AC (1976). Zoogeographic patterns in chaetognaths and some other planktonic organisms.. Bulletin Zoologisch Museum Universiteit van Amsterdam.

[18] Hebert PDN, Alina C, Shelley LB, Jeremy RD (2003). Biological identifications through DNA barcodes.. Proceedings of the Royal Society of London Series B: Biological Sciences.

[19] Hajibabaei M, Janzen DH, Burns JM, Hallwachs W, Hebert PDN (2006). DNA barcodes distinguish species of tropical Lepidoptera.. Proceedings of the National Academy of Sciences USA.

[20] Clare EL, Lim BK, Engstrom MD, Eger JL, Hebert PDN (2007). DNA barcoding of Neotropical bats: species identification and discovery within Guyana.. Molecular Ecology Notes.

[21] Pfenninger M, Nowak C, Kley C, Steinke D, Streit B (2007). Utility of DNA taxonomy and barcoding for the inference of larval community structure in morphologically cryptic *Chironomous* (Diptera) species.. Molecular Ecology.

[22] Ward RD, Zemlak TS, Innes BH, Last PR, Hebert PDN (2005). DNA barcoding Australia's fish species.. Philosophical Transactions of the Royal Society of London, Series B, Biological Sciences.

[23] Kelly RP, Sarkar IN, Eernisse DJ, DeSalle R (2007). DNA barcoding using chitons (genus *Mopalia*).. Molecular Ecology Notes.

[24] Costa FO, deWaard JR, Boutillier J, Ratnasingham S, Dooh RT (2007). Biological identifications through DNA barcodes: the case of the Crustacea.. Canadian Journal of Fisheries and Aquatic Science.

[25] Papillon D, Perez Y, Caubit X, Le Parco Y (2006). Systematics of Chaetognatha under the light of molecular data, using duplicated ribosomal 18S DNA sequences.. Molecular Phylogenetics and Evolution.

[26] Pleijel F, Jondelius U, Norlinder E, Nygren A, Oxelman B (2008). Phylogenies without roots? A plea for the use of vouchers in molecular phylogenetic studies.. Molecular Phylogenetics and Evolution.

[27] Folmer O, Black M, Hoeh W, Lutz R, Vrijenhoek R (1994). DNA primers for amplification of mitochondrial cytochrome c oxidase subunit I from diverse metazoan invertebrates.. Molecular Marine Biology and Biotechnology.

[28] Altschul SF, Gish W, Miller W, Myers EW, Lipman DJ (1990). Basic local alignment search tool.. Journal of Molecular Biology.

[29] Larkin MA, Blackshields G, Brown NP, Chenna R, McGettigan PA (2007). Clustal W and Clustal X version 2.0.. Bioinformatics.

[30] Kimura M (1980). A simple method for estimating evolutionary rate of base substitution through comparative studies of nucleotide sequences.. Journal of Molecular Evolution.

[31] Tamura K, Dudley J, Nei M, Kumar S (2007). MEGA4: Molecular Evolutionary Genetics Analysis (MEGA) software version 4.0.. Molecular Biology and Evolution.

[32] Nylander JAA (2004). MrModeltest v2..

[33] Ronquist F, Huelsenbeck JP (2003). MRBAYES 3: Bayesian phylogenetic inference under mixed models.. Bioinformatics.

[34] Guindon S, Gascuel O (2003). A simple, fast, and accurate algorithm to estimate large phylogenies by maximum likelihood.. Systematic Biology.

[35] Guindon S, Lethiec F, Duroux P, Gascuel O (2005). PHYML Online—a web server for fast maximum likelihood-based phylogenetic inference.. Nucleic Acids Research.

[36] Anisimova M, Gascuel O (2006). Approximate likelihood-ratio test for branches: A fast, accurate, and powerful alternative.. Systematic Biology.

[37] Waugh J (2007). DNA barcoding in animal species: progress, potential and pitfalls.. BioEssays.

[38] Peijnenburg K, van Haastrecht E, Fauvelot C (2005). Present-day genetic composition suggests contrasting demographic histories of two dominant chaetognaths of the North-East Atlantic, *Sagitta elegans* and *S. setosa*.. Marine biology.

[39] Telford MJ, Holland PWH (1997). Evolution of 28S ribosomal DNA in chaetognaths: Duplicate genes and molecular phylogeny.. Journal of Molecular Evolution.

